# Feasibility of Measuring Screen Time, Activity, and Context Among Families With Preschoolers: Intensive Longitudinal Pilot Study

**DOI:** 10.2196/40572

**Published:** 2022-09-29

**Authors:** Hannah Parker, Sarah Burkart, Layton Reesor-Oyer, Michal T Smith, Roddrick Dugger, Lauren von Klinggraeff, R Glenn Weaver, Michael W Beets, Bridget Armstrong

**Affiliations:** 1 Department of Exercise Science Arnold School of Public Health University of South Carolina Columbia, SC United States

**Keywords:** ecological momentary assessment, accelerometry, objective digital media use, screen time, sleep, activity, preschool, dyads, mobile phone

## Abstract

**Background:**

Digital media has made screen time more available across multiple contexts, but our understanding of the ways children and families use digital media has lagged behind the rapid adoption of this technology.

**Objective:**

This study evaluated the feasibility of an intensive longitudinal data collection protocol to objectively measure digital media use, physical activity, sleep, sedentary behavior, and socioemotional context among caregiver-child dyads. This paper also describes preliminary convergent validity of ecological momentary assessment (EMA) measures and preliminary agreement between caregiver self-reported phone use and phone use collected from passive mobile sensing.

**Methods:**

Caregivers and their preschool-aged child (3-5 years) were recruited to complete a 30-day assessment protocol. Within 30-days, caregivers completed 7 days of EMA to measure child behavior problems and caregiver stress. Caregivers and children wore an Axivity AX3 (Newcastle Upon Tyne) accelerometer to assess physical activity, sedentary behavior, and sleep. Phone use was assessed via passive mobile sensing; we used Chronicle for Android users and screenshots of iOS screen time metrics for iOS users. Participants were invited to complete a second 14-day protocol approximately 3-12 months after their first assessment. We used Pearson correlations to examine preliminary convergent validity between validated questionnaire measures of caregiver psychological functioning, child behavior, and EMA items. Root mean square errors were computed to examine the preliminary agreement between caregiver self-reported phone use and objective phone use.

**Results:**

Of 110 consenting participants, 105 completed all protocols (105/110, 95.5% retention rate). Compliance was defined a priori as completing ≥70%-75% of each protocol task. There were high compliance rates for passive mobile sensing for both Android (38/40, 95%) and iOS (64/65, 98%). EMA compliance was high (105/105, 100%), but fewer caregivers and children were compliant with accelerometry (62/99, 63% and 40/100, 40%, respectively). Average daily phone use was 383.4 (SD 157.0) minutes for Android users and 354.7 (SD 137.6) minutes for iOS users. There was poor agreement between objective and caregiver self-reported phone use; root mean square errors were 157.1 and 81.4 for Android and iOS users, respectively. Among families who completed the first assessment, 91 re-enrolled to complete the protocol a second time, approximately 7 months later (91/105, 86.7% retention rate).

**Conclusions:**

It is feasible to collect intensive longitudinal data on objective digital media use simultaneously with accelerometry and EMA from an economically and racially diverse sample of families with preschool-aged children. The high compliance and retention of the study sample are encouraging signs that these methods of intensive longitudinal data collection can be completed in a longitudinal cohort study. The lack of agreement between self-reported and objectively measured mobile phone use highlights the need for additional research using objective methods to measure digital media use.

**International Registered Report Identifier (IRRID):**

RR2-36240

## Introduction

### Background

Excessive screen time for children is linked with poor sleep, inactivity, and behavior problems [1-4]. Few preschool-aged children meet the World Health Organization’s recommendation ≤1 hour of screen time per day [5,6]. This is partly attributable to the rapid growth of digital media technology, which makes screens more available across multiple contexts [7,8]. Unfortunately, our understanding of the unique ways in which children and families use digital media has lagged behind the rapid adoption of this technology. Therefore, we need updated paradigms to understand how families use digital media and how it impacts their health.

Research on digital media use (defined here as tablet or mobile phone use) has been hindered by methodological limitations, including a reliance on retrospective self-reported measures (which are subject to recall and desirability biases) [9-11] and a tendency to overlook momentary etiological processes occurring within each day [10]. The limited research on within-day time-varying contexts (ie, child behavior and parenting stress) that influence digital media use in specific situations leads to a coarse understanding of the mechanisms underlying the link between screen time and behavioral health outcomes.

Parents allow children to use digital media for a variety of reasons (eg, to provide relief from caregiving, and to modify behavior) [12], and the decisions parents make regarding how and when they use digital media must balance the immediate needs of themselves and their children. The parent-child processes around digital media use are likely bidirectional, where child factors (ie, behavior problems) and digital media habits influence each other through a transactional process. For example, parents might use digital media to soothe or distract a fussy child [13], which reduces the frequency of enriching parent-child interactions [14-16] and contributes to continued behavioral difficulties [17,18], which in turn predict greater digital media exposure. The importance of understanding behavioral patterns that vary across contexts and families lies in the ability to identify the most salient causal pathways that can be used to develop individualized, tailored, and targeted intervention strategies [19]. Accurate and acceptable measurement of digital media use is necessary to elucidate the potentially unique mechanisms underlying digital media use and health behaviors.

Technological advances in passive mobile sensing have made it possible to access data already collected by mobile devices (ie, app use) to objectively measure digital media use [20,21]. By integrating intensive longitudinal data collected from multiple sources (ie, passive mobile screen time sensing, ecological momentary assessment [EMA], and accelerometry), we can reveal the process-oriented science that underlies the association between mobile phone use and other health behaviors (ie, sleep and activity) and socioemotional health (ie, behavior problems) [22]. To our knowledge, no studies to date have leveraged a combination of EMA, accelerometry, and passive mobile sensing capabilities to specifically study digital media use among families with young children [11,23]. It is unclear whether this combination of methods can be successfully used to gather meaningful information about the digital media use of children and families.

### Objectives

Feasible and accurate measures of digital media use and context are a necessary first step toward understanding the dynamics of children’s digital media use [10]. Therefore, this study aimed to evaluate the feasibility of a 2-wave intensive longitudinal data collection protocol designed to measure digital media use (ie, mobile phones and tablets), physical activity, sleep, sedentary behavior, and socioemotional context among caregiver-child dyads. The entire study protocol was administered twice (both instances being an average of 7 months apart) to examine the feasibility of recruiting and retaining a cohort willing to complete the protocol multiple times. Additional aims of this study were to describe the convergent validity of EMA measures and to describe preliminary agreement between caregiver self-reported digital media use and mobile phone use collected from passive mobile sensing.

## Methods

### Study Design

This pilot intensive longitudinal study aimed to recruit 100 caregiver-child dyads. The study used an observational case-crossover design [24], where dyads served as their own controls to assess the within- and between-day effects of immediate antecedents on a dependent variable measured multiple times throughout the day and week [24]. Caregivers and their 3- to 5-year-old children participated in a 30-day assessment protocol. The sample was invited back approximately 3-12 months after their initial assessment (depending on initial recruitment date) to complete a modified 14-day protocol.

### Ethical Considerations

The study protocol was approved by the institutional review board at the University of South Carolina in August 2020 (Pro00092634). Owing to COVID-19 protocol adjustments [25], consent was obtained remotely. Interested participants were directed to an informational website that described the study procedures and participant rights and protections and included a web-based consent form. Following the web-based consent process, eligible participants were contacted by a trained member of the research team by phone to verbally explain study procedures, answer questions, and confirm their desire to participate.

### Recruitment

Recruitment took place between September 2020 and September 2021. Recruitment and enrollment procedures are described elsewhere [26]. Briefly, we recruited a nonrandom volunteer sample by distributing flyers at daycare centers, pediatric clinics, and community centers. We also created boosted Facebook posts. In an effort to recruit a socioeconomically and racially diverse sample, we reached out directly to daycares serving low-income families and prioritized the enrollment of low-income families. Finally, we used a snowball recruitment strategy and compensated participants US $10 if they referred another eligible family to participate in the study and if the referred family completed the protocol.

### Participants

Caregivers were eligible if they (1) were a primary caregiver of a child aged between 3 and 5 years (ie, preschool aged), (2) owned a smartphone device, and (3) were able to read and speak English. Exclusion criteria for children included a diagnosis of a severe developmental or physical disorder that would prevent ambulation.

### Study Protocol

#### Overview

Study procedures are described in detail elsewhere [26]. Generally, the day-to-day operations of the study included participant communication, participant enrollment, and participant tracking. Daily operations were predominately conducted by 1 postdoctoral fellow, 1 doctoral student, and 1 staff member, with oversight from the principal investigator.

#### First Wave of Data Collection

Briefly, after consenting, caregivers completed a baseline survey that contained measures of demographics, parental stress, screen time, psychological functioning, and child behavior. Caregivers completed EMAs for 7 days to measure child behavior problems, caregiver stress, and child screen time. Each day 4 signal-contingent EMAs were delivered to caregivers’ mobile phones between 8:30 AM and 9 PM. The surveys expired after 2 hours and were delivered such that no overlapping time windows could exist. EMA assessments were conducted in the first week of the 30-day monitoring window. Additional details, including a full list of EMA questions, can be found elsewhere [26]. In addition, caregivers and children were asked to wear an Axivity AX3 accelerometer (Newcastle upon Tyne, United Kingdom) on their nondominant wrist for 24 hours per day (including while bathing and swimming) for 30 days to assess physical activity, sedentary behavior, and sleep. Nondominant wrist placement has been shown to improve compliance rates compared with waist placement [27]. Data were processed using GGIR (version 2.5.1) [28] in R (R Foundation for Statistical Computing). Mobile phone use over 30 days differed based on whether the caregiver used an iOS or an Android device. For participants with an Android device, we used Chronicle [29], an app designed specifically for passive screen time monitoring of Android devices. Caregivers were sent a link to download the app and asked to allow it to run on their phone during the study period and delete it after 30 days. Caregivers with an iOS phone were texted an automated reminder to send a screenshot of their screen time use each day at 9 PM to a study-specific phone number. If a caregiver failed to send a correct screenshot during the 7 days of EMAs, study staff sent a personalized SMS text message within 24 hours. After the 7-day EMA period, study staff sent a personalized SMS text message if a caregiver failed to send a screenshot on 2 consecutive days (48 hours). Participants were compensated up to US $180 for their participation in each wave of data collection (US $360 in total; see [26] for more details). The number of reminders to send images was recorded as an indicator of feasibility. The use of technical support (participant use of research staff technical support as well as research staff requests from Chronicle support staff) was included as a screen time feasibility outcome. We attempted to conduct semistructured qualitative interviews with all participants who dropped out of the study.

#### Second Wave of Data Collection

Approximately 3-12 months after their initial enrollment, families were invited to participate in the entire study protocol for a second time. The second wave was conducted to examine the feasibility of retaining a cohort sample and to assess the longer-term acceptability of the protocol in its entirety. Several protocol adjustments were made between waves 1 and 2 based on participant feedback from wave 1. Most notably, the monitoring period for EMA was increased from 7 to 14 days, and accelerometry and passive mobile sensing were shortened from 30 to 14 days to align with the EMA protocol. Logistically, shortening the accelerometer protocol allowed us to invite all participants to re-enroll in the second wave of data collection (only 50/100, 50% was initially proposed). In addition, *Qustodio* was used to passively monitor iOS devices (phones and tablets) and Amazon Kindle Fire tablets (a description of Qustodio is provided in the following section).

During the second wave of data collection, the study team began using Qustodio, a commercially available passive sensing app that allows a third party (ie, parents) to monitor the timing, duration, and content of digital media. Starting in October 2021, caregivers were invited to allow the research team to monitor their child’s tablets (Kindle and iOS devices) using the media monitoring system, Qustodio. Starting in January 2022, caregivers with iOS devices were also asked to download Qustodio for passive mobile sensing instead of uploading screenshots, as was done in wave 1. To monitor devices with Qustodio, caregivers were sent a link to download the app. Participants with iOS devices were sent additional instructions to install a mobile configuration, as iOS did not allow third-party apps to monitor screen use directly. A research assistant worked with caregivers over the phone to complete the enrollment. Unlike Chronicle, each Qustodio enrollment required direct assistance from a member of the research team via call or text messaging (approximately 10 minutes per participant). Caregivers were instructed to delete the app and mobile configuration and unenroll after the study period (14 days). Enrollment of a child device was presented as optional for additional compensation, and the number of caregivers who agreed to enroll, number of successful enrollments, and number of days of data were collected as feasibility metrics.

### Primary Outcome Measures

The specific feasibility outcome measures for this pilot study were as follows:

- Recruitment rate was operationalized as the number of eligible families divided by the total number of families interested.
- Enrollment rate was defined as the number of participants enrolled divided by the number of participants approached to participate.
- Retention rate was defined as the number of participants who completed the study protocol (ie, engaged in each study task) divided by the total number of consenting and enrolled participants.
- Re-enrollment rate was defined as the number of participants who re-enrolled in the second wave of data collection divided by the number of participants who completed the initial wave of data collection.
- Mobile phone compliance was defined a priori as having 21 out of 30 (wave 1) and 10 out of 14 (wave 2) days of screen time data. For iOS users, this entailed texting at least 21 (wave 1) screen-use images and 10 (wave 2) screen-use images to research study staff or 10 out of 14 days of successfully monitored data using the Qustodio software. For Android users, this entailed having at least 21 (wave 1) and 10 (wave 2) days of successfully monitored data using the Chronicle software. Screen time compliance did not include participants who were unable to comply because of Technological issues (refer to the Technological Issues section). Participants were not offered an extension beyond 30 days to reach compliance.
- Accelerometer compliance was defined a priori as at least 21 of 30 days of valid accelerometer data for wave 1 and 10 of 14 days of valid accelerometer data for wave 2. A valid day of accelerometer data was defined as at least 16 hours of wear during a 24-hour period.
- EMA compliance was defined a priori as completing 21 out of 28 surveys for wave 1 and 39 out of 56 surveys for wave 2. We calculated the number of participants who reached strict compliance (wave 1: 21 surveys within a 7-day window and wave 2: 39 surveys within a 14-day window). Participants who were unable to meet strict compliance criteria within the designated window were offered an extension to reach compliance (not exceeding 30 days). The number of additional days needed to reach 21 surveys was also recorded.

### Secondary Outcome Measures

#### Measurement Validity

In addition to testing protocol feasibility, we aimed to examine preliminary convergent and divergent validity of EMA measures of child behavior and parental stress with established measures of each construct, which were administered once at the beginning of the study period. We also aimed to examine the agreement between passive mobile phone sensing compared with a self-report questionnaire measure of mobile phone use, which is the current standard in many screen-use studies [30,31].

#### Child Behavior

A full list of EMA items is described elsewhere [26]. Items were adapted from the Multidimensional Assessment of Preschool Disruptive Behavior scale [32] and assessed child tantrums, noncompliance, and aggression over the previous 2 hours. Behavior problems were aggregated at the person level and calculated as the count of EMA completions where caregivers endorsed a problem behavior divided by the total number of potential EMA completions when caregivers and children were together. EMA-measured behavior problems were compared with subscales on the Strengths and Difficulties Questionnaire (SDQ) [33], which was completed once during the baseline survey before the start of the EMA protocol.

#### Caregiver Stress

Caregiver stress was assessed by EMA using 2 items used in previous EMA studies [34,35]. The items were “How stressed are you feeling right now?” and “How certain do you feel that you can deal with all the things that you have to do right now?” The items were rated on a 5-point and 4-point Likert scale, respectively, from “1 (not at all)” to “4 or 5 (extremely),” and the items were aggregated for each participant across all EMA time points. We then examined associations between EMA-measured stress and validated measures of stress as well as parental distress and psychological functioning, both of which were constructs hypothesized to be related to stress. Measures were completed once during the baseline survey before the start of the EMA protocol. The measured constructs included overall stress (assessed using the Perceived Stress Scale [36]) as well as caregiver distress (assessed using the Kansas Parental Satisfaction Scale [37] and Confusion, Hubbub, and Order Scale [CHAOS] [38]) and psychological functioning (assessed using the Center for Epidemiologic Studies-Depression [39] and the short-form State-Trait Anxiety Inventory [40]).

#### Objective Phone Use (Passive Mobile Sensing)

We examined the preliminary agreement between passive mobile sensing screen time estimates compared with an existing widely used self-report questionnaire measure of technology use [41]. Specifically, caregivers answered the prompt “Thinking of an average weekday/weekend day (from when you wake up until you go to sleep), how much time do you spend using a smartphone as the primary activity?” Prompts for weekends and weekdays were assessed separately. For analyses, a composite measure was created that weighted weekend and weekday responses for an average daily use estimate. Notably, although this measure has shown good reliability, research on validity of self-report mobile phone use measures is largely absent from the literature. Thus, we refrain from using the term “convergent validity” when describing the associations between passive mobile sensing and self-report measures.

*Technological issues* with screen time monitoring were recorded and described. For iOS users, we monitored the number of days that required a personalized reminder prompt from the study team to send the image the following day. For Android (Chronicle), we tracked the number and type of technological complications as well as the number of participants impacted for the first 18 Android participants (between September 8, 2020, and July 7, 2021). The number of technological issues was only tracked for a subset of the study participants because of a change in help desk request procedures for Chronicle. Caregivers with an Android device were provided with instructions to enroll in Chronicle, an app designed specifically for passive screen time monitoring on Android devices [29].

### Protocol Modifications and Post Hoc Analyses

In this section, we describe protocol adjustments and modifications made over the course of the study. As these were not anticipated before the start of the study, we have elected to present them as post hoc and opportunistic analyses.

**Figure 1:**
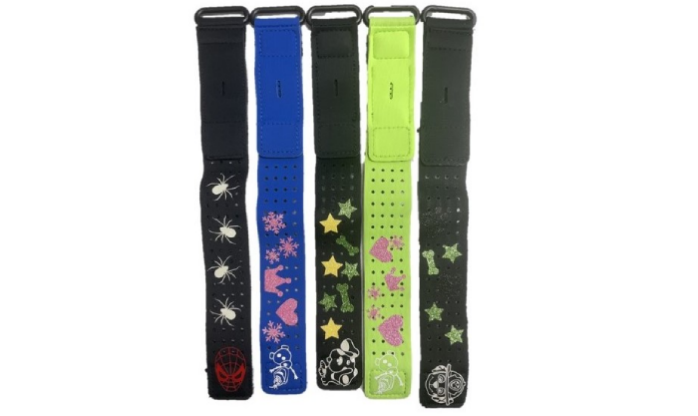
Decorated bands for child accelerometers.

In response to low initial compliance rates for child accelerometer wear, we examined options to increase compliance. In this opportunistic nonrandomized subanalysis, the first 30 children recruited into the study were sent an undecorated accelerometer band, after which wear compliance was preliminarily examined. In an effort to improve compliance, the next 75 children enrolled were sent decorated bands featuring iron-on decals of characters from Frozen, PAW Patrol, Marvel, or Blue’s Clues, depending on the child’s preferred characters (Figure 1).

### Statistical Analysis

All data were analyzed using SPSS (version 27; IBM Corp) and Stata SE (version 16.1; StataCorp). In this section, we present descriptive statistics of feasibility outcomes. Bayesian Pearson correlations (with a 95% credible interval) were used to assess the association between EMA problem behavior and SDQ subscales. Independent samples *t* tests (2-tailed) were used to examine differences between participants who completed the protocol and those who dropped out. Root mean square errors (RMSEs) were calculated to examine the agreement between parent-reported mobile phone use and objectively measured mobile phone use. As feasibility is the primary outcome of interest in this study, we examined the signal of effect difference using standard effect size estimates (ie, Cohen *d* and *r*) and minimal acceptable feasibility metrics in favor of significance testing [42]. For post hoc tests of protocol additions, we conducted 2-tailed independent samples *t* tests to compare valid days of wear between participants who received decorated accelerometer bands and participants who received undecorated accelerometer bands.

### Sample Size

Given that this is a pilot study, no power analysis was required [43].

## Results

### Sample Characteristics

**Figure 2:**
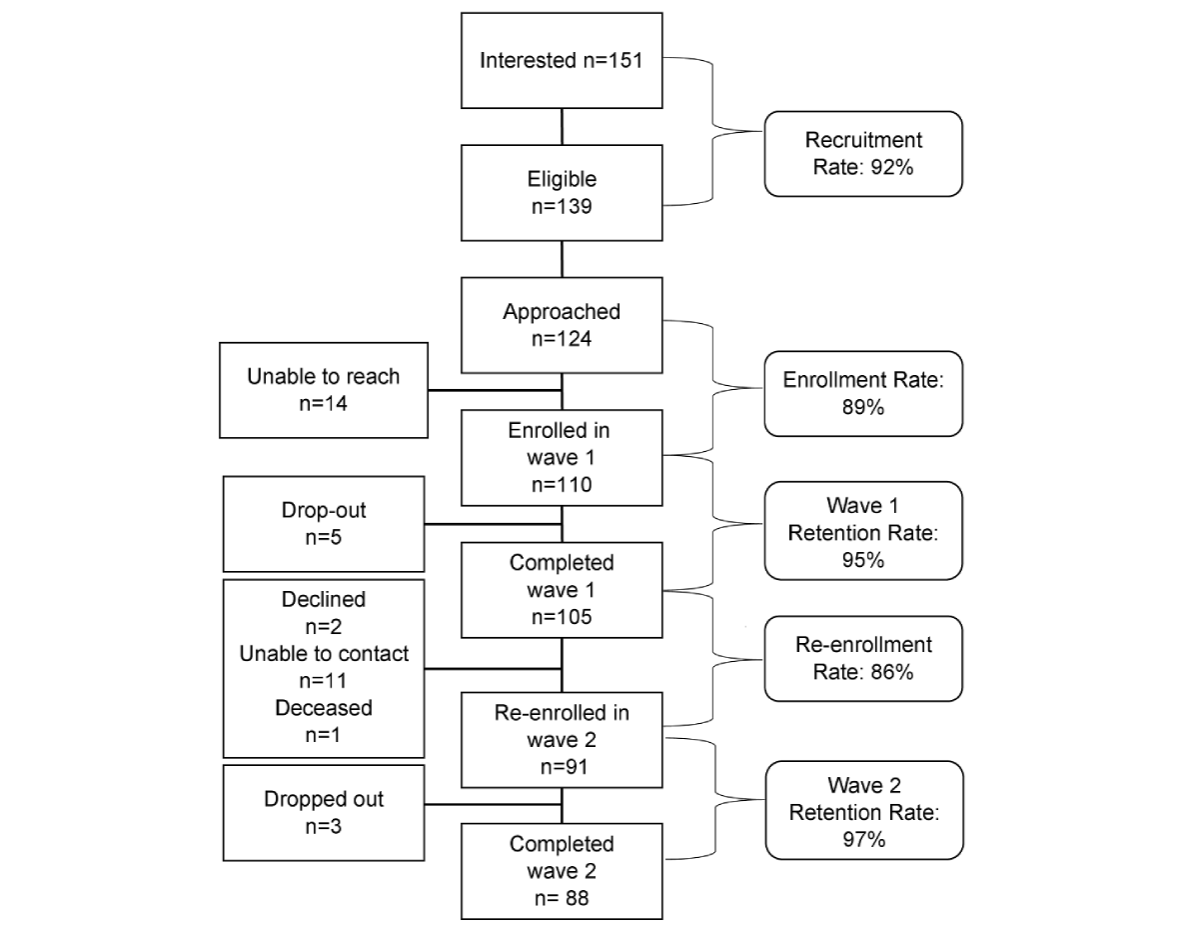
Flowchart showing recruitment and retention of study participants.

The flow of participants through the study is presented in Figure 2. Demographics of participants at wave 1 are presented in Table 1.

**Table 1:** Participant demographics (N=105).

Characteristics				Values
**Child demographics**				
	Age (years), mean (SD)		4.4 (0.8)	
	**Race, n (%)**			
		African American	32 (30)	
		White	66 (63)	
		Other	7 (7)	
	Hispanic ethnicity, n (%)		4 (4)	
	Female sex, n (%)		51 (49)	
	**Child device^a^, n (%)**			
		None	16 (15)	
		Android	30 (29)	
		iOS	32 (30)	
		Kindle Fire	28 (27)	
		Other	9 (9)	
		Unknown or unable to verify	5 (5)	
**Caregiver demographics**				
	Age (years; range 22-78), mean (SD)		36.6 (7.8)	
	Female sex, n (%)		100 (95)	
	**Relationship with child, n (%)**			
		Mother	95 (90)	
		Father	6 (6)	
		Grandparent	3 (3)	
		Other	1 (1)	
	**Phone type, n (%)**			
		iPhone	65 (62)	
		Android	40 (38)	
	**Education, n (%)**			
		Less than high school	1 (1)	
		High school	8 (8)	
		Some college or vocational training	25 (24)	
		2-year degree	13 (12)	
		4-year degree	31 (30)	
		Doctorate or professional degree	27 (26)	
	**Social assistance programs, n (%)**			
		WIC^b^	25 (24)	
		SNAP^c^	28 (27)	
		Medicaid	48 (46)	
	**Income (US $), n (%)**			
		<20,000	14 (13)	
		20,000-40,000	19 (18)	
		40,000-60,000	28 (27)	
		60,000-80,000	12 (11)	
		80,000-100,000	13 (12)	
		>100,000	19 (18)	

^a^Child could have more than 1 device, so the percentages do not add up to 100%.

^b^WIC: Special Supplemental Nutrition Program for Women, Infants, and Children.

^c^SNAP: Supplemental Nutrition Assistance Program.

### Primary Outcomes

Primary recruitment, enrollment and re-enrollment, and retention rates are presented in Figure 2. Protocol compliance is presented in Table 2. All sample recruitment, retention, and enrollment rates were >85%. Retention between the first and second waves of data collection was 86.7% (91/105). Participants were re-enrolled an average of 7.3 (SD 2.8) months after their initial enrollment. Outcomes are presented separately for wave 1 and wave 2.

**Table 2:** Compliance rates for primary outcome measures.

Outcome measure			Wave 1						Wave 2				
			Compliant/possible n		Participants meeting compliance criteria (%)		Number of valid observations, mean (SD; range)		Compliant/possible n		Compliant (%)		Number of valid observations, mean (SD; range)
**Accelerometry^a^**													
	Parent^b^	62/99		63		19.4 (7.5; 0-27)		72/84		86		13.4 (4.0; 0-22)	
	Child^c^	40/100		40		15.5 (8.2; 0-28)		57/85		67		10.8 (5.3; 0-20)	
**Mobile phone^a^**													
	Android	38/40		95		32.0 (7.6; 7-53)		30/32		94		16.8 (4.8; 2-29)	
	iPhone	64/65		98		28.9 (2.9; 18-37)		54/56		96		16.3 (4.4; 0-30)	
	EMA^d,^^e^	105/105		100		26.2 (2.2; 21-32)		85/88		97		48.6 (6.9; 19-60)	

^a^Wave 1 compliance was defined as 21 days of valid data provided; wave 2 compliance was defined as 9 days of valid data provided.

^b^Wave 1: lost device=1 and battery failure=5; wave 2: lost device=2 and battery failure=2.

^c^Wave 1: lost device=4 and battery failure=1; wave 2: lost device=3.

^d^EMA: ecological momentary assessment.

^e^Wave 1 compliance was defined as 21 prompts answered; wave 2 compliance was defined as 39 prompts answered.

### Wave 1

#### Mobile Phone Monitoring Compliance

Screen time compliance is presented separately for Android and iOS devices, given the different data collection protocol procedures. A majority of both Android (38/40, 95%) and iOS (64/65, 98%) users met the study definition of compliance (≥21 days of data). Notably, the remaining 5% (2/40) of Android participants did not meet the criteria because of technological issues with Chronicle, not study-related noncompliance. In all, 47.6% (50/105) of participants had over 30 days of data collection, attributable to the following reasons: (1) participants enrolling in screen time monitoring (Android) before receiving the activity watches and starting the protocol, (2) participants failing to delete the Chronicle app, or (3) participants sending additional screenshot images even after the end of their study observation period. The mean number of valid days of observation data were 32.0 (SD 7.6) and 28.9 (SD 2.9) among Android and iOS users, respectively.

#### Technological Issues

Technological issues emerged among both iOS and Android users. For iOS users, reminder prompts were sent on 32 occasions across 18 participants. Issues for iOS users were parents forgetting to send their nightly screenshots without an additional reminder from research study staff. For Chronicle, for the first 18 participants (September 2020 through July 7, 2021), there were 16 reports of technological issues. Issues for Android users were related to data processing problems with Chronicle, which were beyond the immediate control of the research staff (Table 3). Technological issues included (1) battery issues that occurred when the participant had battery optimization turned on their smartphones, (2) server malfunctioning, (3) large data sets causing data upload failures, and (4) data not uploading to the cloud from Chronicle.

**Table 3:** Technological issues.

			Number of incidents		Number of participants impacted/number of participants possible (%)
**Chronicle (Android)^a^**					
	Battery issues	6		6/18 (33)	
	Processing issues	11		11/18 (61)	
**iOS (iPhone)**					
	Number of reminder prompts sent	32		18/41 (44)	

^a^Data were tracked between September 8, 2020, and July 7, 2021, across 18 Android participants.

#### Accelerometer Compliance

Children wore the accelerometer for an average of 15.5 (SD 8.2) days during the 30-day monitoring period. A total of 4 children lost their device, and 1 device was returned with a battery malfunction. Among the children who returned a working device, 40% (40/100) met the study-specific compliance criteria of ≥21 days of data. Furthermore, 3 children returned the device but had 0 days of valid data. However, 91% (91/100) of the children had at least three days of valid data, a common criterion used in studies of children’s physical activity [44].

Caregivers wore the accelerometer for an average of 19.4 (SD 7.5) days during the 30-day monitoring period*.* One caregiver lost the device, and 5 devices were returned with battery malfunctions. Among parents who returned a working device, 63% (62/99) met the study-specific compliance criteria of ≥21 days of data. In all, 96% (95/99) caregivers wore the device for at least three days.

#### EMA Compliance

EMA compliance was high, with 100% (105/105) of caregivers completing at least 21 EMAs. EMAs were completed in an average of 2.8 (SD 10.24) minutes. In all, 95% of all EMA prompts were completed in ≤5 minutes. In all, 9.5% (10/105) of caregivers required additional opportunities to reach 21 completions. Of those who were provided with additional opportunities, caregivers were sent an average of 11 (SD 7.8) additional EMAs. Most participants (95/105, 90.5%) completed at least 21 EMAs, with no additional opportunities. Overall, caregivers completed an average of 26.2 (SD 2.2) total EMAs.

#### Dropouts

There were minimal demographic differences between dropouts (5/110, 4.5%) and those who completed the protocol in terms of child age (Cohen *d*=−0.1; 95% CI −1.1 to 0.9), caregiver age (Cohen *d*=0.9; 95% CI −0.1 to 1.9), or employment status (Cohen *d*=0.3; 95% CI −0.7 to 1.3). The largest difference was observed for income (Cohen *d*=1.8; 95% CI 0.3-2.4), where participants who dropped out had lower incomes. One participant consented but never completed any study procedures. Of the 5 families who started the protocol and then dropped out, none of the caregivers agreed to participate in a semistructured qualitative interview about their experience. However, a review of text correspondence with project staff indicated that reasons for dropping out were lack of time (2/5, 40%) and no longer wanting to participate (1/5, 20%). The remaining 40% (2/5) of participants stopped responding to study-related SMS text messages.

### Wave 2

#### Mobile Phone Monitoring Compliance

Screen time compliance is presented separately for Android and iOS devices, given the different data collection protocol procedures. A majority of both Android (30/32, 94%) and iOS (54/56, 96%) users met the study definition of compliance (≥10 days of data). In all, 81% (71/88) of participants had over 14 days of data collection, attributable to the following reasons: (1) participants enrolling in screen time monitoring before receiving the activity watches and starting the protocol, (2) failing to delete the screen time monitoring app (Chronicle or Qustodio), (3) sending additional screenshot images after the end of the observation period, or (4) extending the study period to have extra EMA opportunities. The mean number of valid days of observation data were 16.8 (SD 4.8) and 16.3 (SD 4.4) among Android and iOS users, respectively.

#### Accelerometer Compliance

Children wore the accelerometer for an average of 10.8 (SD 5.3) days during the 14-day monitoring period. A total of 3 children lost their device. Among the children who returned a device, 67% (57/85) met the wave 2 study-specific compliance criteria of ≥10 days of data. Another 5% (4/85) of children returned the device but had 0 days of valid data. However, 87% (74/85) of the children had at least 3 days of valid data, a common criterion used in studies of children’s physical activity [44]. Caregivers wore the accelerometer for an average of 13.4 (SD 4.0) days during the 14-day monitoring period*.* A total of 2 caregivers lost the device, and 2 devices were returned with a battery malfunction. One caregiver returned the device with 0 days of valid data. In all, 96% (81/84) of caregivers wore the device for at least three days, and 86% (72/84) of caregivers met the study-specific compliance criteria of ≥10 days.

#### EMA Compliance

EMA compliance was high, with 97% (85/88) of caregivers completing at least 39 EMAs. In all, 10% (9/88) of caregivers were provided with extra EMA opportunities to reach the compliance threshold. Of those who were provided with additional opportunities, caregivers were sent an average of 9 (SD 10.1) additional EMAs. Most participants (79/88, 90%) completed at least 39 EMAs with no additional opportunities. Overall, caregivers completed an average of 48.6 (SD 6.9) EMAs.

### Secondary Outcomes

#### Child Behavior

**Figure 3:**
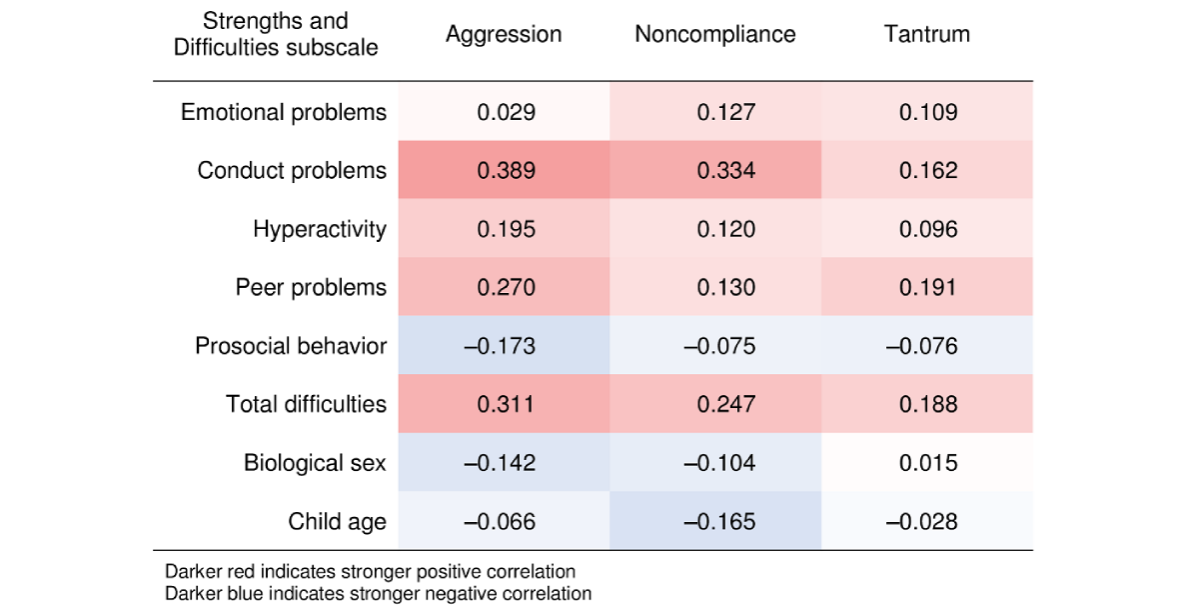
Correlations between Strengths and Difficulties subscales and ecological momentary assessment (EMA) behaviors of aggression, noncompliance, and tantrums.

Correlations are presented in Figure 3. Means and SDs are presented in Multimedia Appendix 1. Caregivers reported that their child’s behavior was “a little bit” to “a great deal” problematic in 31% (501/1614) of EMA instances. The most frequent problem behaviors were noncompliance (243/1614, 15.05%), tantrums (174/1614, 10.78%), and aggression (71/1614, 4.39%). For convergent validity, EMA aggression was correlated with SDQ subscales of conduct problems (*r*=0.389; 95% CI 0.219-0.539), total difficulties (*r*=0.311; 95% CI 0.140-0.479), peer problems (*r*=0.270; 95% CI 0.092-0.439), and hyperactivity (*r*=0.195; 95% CI 0.013-0.372). EMA noncompliance was correlated with conduct problems (*r*=0.334; 95% CI 0.165-0.499) and total difficulties (*r*=0.247; 95% CI 0.080-0.423). EMA tantrums were associated with peer problems (*r*=0.191; 95% CI 0.008-0.371) and total difficulties (*r*=0.188; 95% CI 0.000-0.361). The prosocial subscale, child age, and sex, were not strongly associated with aggression (*r=*−0.027 to −0.142), noncompliance (*r=*−0.165 to −0.002), or tantrums (*r*=−0.095 to 0.015).

#### Caregiver Stress

**Figure 4:**
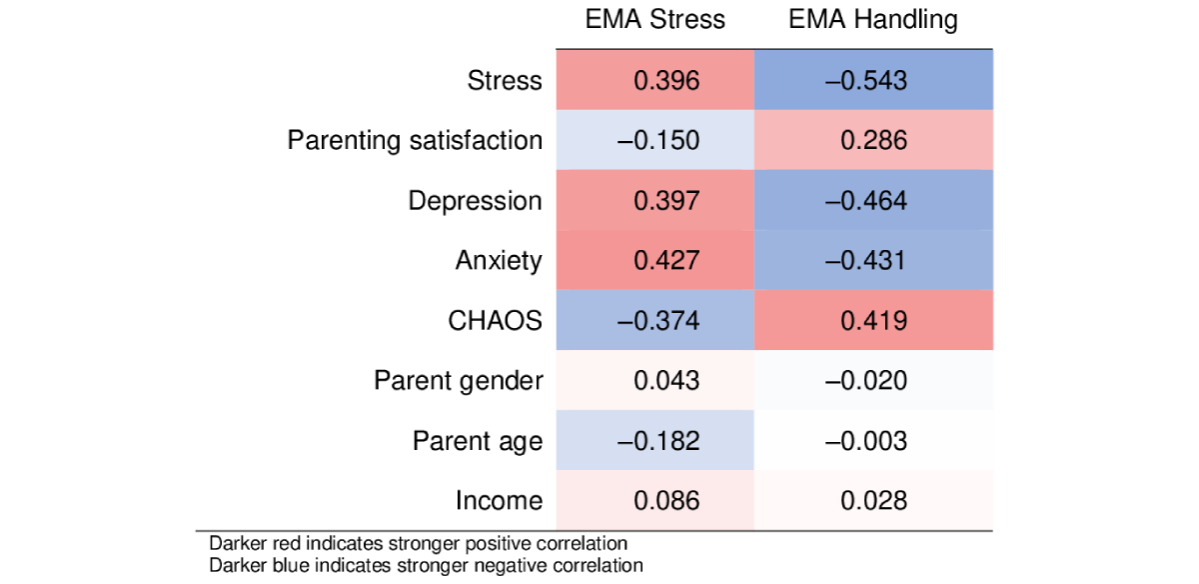
Correlations between ecological momentary assessment (EMA) measures of stress and established measures of stress, parenting satisfaction, anxiety, depression, and household chaos. CHAOS: Confusion, Hubbub, and Order Scale - Lower scores indicate greater household disorganization.

Correlations are presented in Figure 4. Means and SDs are presented in Multimedia Appendix 1. EMA items regarding caregiver stress were compared with established measures of stress, parenting satisfaction, anxiety, depression, and household chaos. Caregivers who reported higher levels of stress on average across all EMA measures reported higher levels of overall stress on an established questionnaire (*r*=0.396; 95% CI 0.222-0.542) as well as more anxiety (*r*=0.435; 95% CI 0.277-0.581). Caregivers who reported higher average confidence in handling stress showed an inverse pattern; caregivers who had higher confidence showed less overall stress (*r*=0.543; 95% CI −0.675 to −0.410), anxiety (*r*=−0.431; 95% CI −0.580 to −0.275), and depression (*r*=−0.464; 95% CI −0.613 to −0.323) and greater parenting satisfaction (*r*=0.286; 95% CI 0.108-0.454). Caregivers who reported higher overall stress had more household disorganization (*r*=−0.374; 95% CI −0.531 to −0.212), whereas caregivers who reported high levels of efficacy in being able to manage stress had lower household disorganization (*r*=0.419; 95% CI 0.260-0.563). Average caregiver EMA-reported stress was less correlated with caregiver biological sex (*r*=0.043; 95% CI −0.143 to 0.230), age (*r=*−0.182; 95% CI −0.360 to −0.007), or income (*r*=0.086; 95% CI −0.107 to 0.267). Similar patterns were observed for efficacy in managing stress.

#### Caregiver Mobile Phone Screen Time

Average daily mobile phone use duration was 383.4 (SD 157.0) minutes for Android participants and 354.7 (SD 137.6) minutes for iOS users. For Android users, agreement with caregiver-reported mobile phone use was poor, with high rates of both underreporting and overreporting compared with self-reported mobile phone use. RMSE for Android was 157.1, indicating that, on average, caregivers misreported their mobile phone use by over 2.5 hours. For iOS users, RMSE was 81.4, indicating that caregivers misreported their mobile phone use by nearly 1.5 hours.

### Protocol Modifications and Post Hoc Analyses

#### Accelerometer Bands

A total of 6 children were excluded because of lost or malfunctioning devices, and 56 children were included in the post hoc analysis. Of 26 nondecorated bands, 11 children wore the Axivity for ≥21 days (42% compliance). Of 30 decorated bands, 18 children wore the Axivity for ≥21 days (60% compliance). Children who received decorated bands had significantly more days of valid wear (mean 19.83, SD 5.56) than those who received undecorated bands (mean 14.38, SD 10.17; t_37.46_=−2.44; *P*=.02; Cohen *d*=8.02). Given these midstudy results, the bands continued to be decorated for the remaining participants.

#### Child Device Enrollment

During the second wave of data collection (starting October 2021), 90 caregiver-child dyads were invited to monitor their children’s devices. Of the 90 caregiver-child dyads invited, 72 (80%) indicated that their child had their own device, 96% (69/72) of which were compatible with Chronicle or Qustodio. Although caregivers from 92% (66/72) of the families agreed to enroll a child device, 88% (63/72) *both* agreed to enroll *and* had a compatible device. Ultimately, 86% (54/63) of families were successfully enrolled. Unsuccessful enrollment was because of the following reasons: technological issues installing the Qustodio app (3/72, 4%), difficulties scheduling a time to enroll the child’s device (3/72, 4%), child device lost (1/72, 1%), caregiver decided not to enroll the child’s device because of lack of use (1/72, 1%), caregiver being unable to access the child’s device (eg, “locked out”; 1/72, 1%). Overall, an average of 16.8 (SD 8.2) days of data per person were captured on child devices using Qustodio and Chronicle.

## Discussion

### Principal Findings

This is the first cohort study to use passive mobile sensing in conjunction with accelerometry and EMA measures with caregiver-child dyads. This preliminary work demonstrates that measures and protocols to collect these multiple streams of data are feasible. The convergent validity between EMA-measured child behavior and caregiver stress shows preliminary validity for using EMA to measure stress and behavior. The generally high levels of compliance and retention of the study sample are encouraging signs that these multiple overlapping methods of intensive longitudinal data can be completed in a longitudinal cohort study. The lack of agreement between self-reported and objectively measured mobile phone use highlights the need for additional objective and low-burden methods of measuring mobile phone use.

Feasibility outcomes revealed that participants were generally compliant with passive mobile sensing but that technology issues resulted in some data loss. It is notable that this technology is still relatively new, and ideally, as this technology becomes more refined, fewer technological issues will arise. Caregivers were generally willing to allow us to monitor their own device, as evidenced by their enrollment in study procedures and high rates of screen time monitoring compliance. Among families where children had their own device, 92% (66/72) of caregivers were willing to let us monitor their children’s device. Although we were only able to collect data on a portion of those devices because of the novelty of this technology of our monitoring software, continued advancement and expanding ease of use of monitoring software would likely increase this number, which bodes well for future studies aiming to examine child digital media use.

Initially, we observed poor accelerometer compliance among children, but this improved following the implementation of a protocol to decorate the accelerometer bands. Notably, the low compliance is likely a function of our strict valid day criteria (ie, 16 hours vs the more commonly used 10 hours) [44] coupled with our relatively high metric of compliance (21 days). Our 21-day compliance is higher than most accelerometer studies, which commonly set compliance at 3 days [44] and use a minimum of 10 hours of wear to determine a valid day. Although not a direct comparison, if we use a criterion of 3 days of valid data (with a 16-hour valid day cutoff), our study shows similar rates of compliance (17% noncompliance) as other studies that use wrist-worn accelerometers (22.7% noncompliance) [45].

This study also demonstrated the feasibility of deploying EMA measures for at least 7 days. Not only did participants complete most prompts but nearly all were also compliant per study protocol definitions and were willing to complete additional days of measurement in subsequent waves of data collection (ie, 14 days). It is worth noting that although 100% (105/105) of the sample were considered compliant using criteria specified a priori, some families needed additional days to accumulate the requisite number of completed EMA prompts. Similar to previous EMA studies [46], this flexibility was built into the protocol to retain participants from diverse and low-income backgrounds who might not be able to meet study requirements within the given time span, given time constraints, resources, acute stressors, or complex life situations. Furthermore, the EMA measures of child behavior and stress used in this study showed preliminary evidence of convergent validity with established validated measures.

Overall, across all study measures, participants reported minimal burden and expressed willingness to continue to participate in future waves. Finally, we were able to recruit and retain a sample to complete measures twice, both instances being an average of 7 months apart, indicating the potential for such methods to be used in cohort studies across development.

### Comparison With Prior Work

Other studies have used EMA among racially and ethnically diverse households [46] but not in conjunction with passive mobile sensing and coupled with both caregiver and child accelerometry. The confluence of these data is necessary to understand how and why screen habits develop and ultimately influence children’s sleep, activity, and socioemotional health. Combining objective digital media use data with microtemporal EMA data will allow us to identify systems of “Granger causality,” which are systems where one behavior (eg, digital media use) predicts future behavior (ie, tantrums). Ensuring that these measures and procedures are both feasible and accurate is a necessary first step toward this goal. The links among behaviors likely vary in direction and magnitude among different individuals. Characterizing these links can guide advanced intervention methods (eg, just-in-time adaptive interventions and continuous tuning interventions) that aim to maximize change in multiple health behaviors.

### Limitations and Next Steps

This study was designed to overcome current measurement limitations regarding mobile phone use. Although our nonrandom sampling approach does represent a threat to external validity (ie, families who volunteer for a study may differ from the general population of families), our successful recruitment and retention of an economically and racially diverse sample speaks to the generalizability of our study findings. In terms of monitoring digital media use, the use of passive sensing represents a significant step forward. However, several key limitations still exist with regard to passive mobile sensing as a method of measuring digital media use. A significant limitation to this work, and to the field at large, is the inability to distinguish who is using a given device (ie, parent, child, or sibling). Although we attempted to ask caregivers when their child was using their smartphone using EMA, these methods were only able to validate to the standard of self-report, which as discussed earlier is inherently flawed. Furthermore, passive mobile sensing is only able to detect whether a screen is on and not whether it is being actively viewed, which in theory could overestimate digital media use. Although some research has attempted to distinguish who is watching a screen based on facial recognition using cameras [47], this work is preliminary and is not currently available for portable digital media. However, although passive mobile sensing is not able to capture all screen use (such as television), this technology represents a significant advancement in the field. Given the recent dramatic increases in children’s use of digital media specifically, and the relatively outdated research on screen time more generally, there is unique value in measuring and investigating digital media use specifically. Research on digital media use specifically (unique from overall screen-use duration) is necessary to understand the unique causes and consequences of this new technology relative to health outcomes. However, low-burden, accurate, and objective measures of digital media use are necessary to advance the science underlying the dynamics and causal factors and outcomes around digital media use.

Although we did not observe any dropouts because of privacy concerns in this study, the issue of data privacy is worth noting. Chronicle was built specifically as a research tool, designed to be Health Insurance Portability and Accountability Act–compliant, with participant protections in mind. Among other features, the dashboard requires secure credentials for separate users, and data are deidentified and not linked to any IP addresses or phone numbers [29]. Participants were informed that Chronicle tracks mobile device use in terms of whether the phone is on or off, which apps are running, and what time of the day the apps are used. Chronicle does not collect personal information, phone contacts, content of any SMS text messages or emails, information on what websites are visited, or other specifics such as which videos are watched on YouTube. In contrast, third-party apps such as Qustodio have capacities that may not be useful or desirable to researchers (eg, the ability to log keystrokes and website blocking capability). Researchers using passive mobile sensing apps should be aware of data security (how data are being stored and transmitted) as well as data ownership (to whom do the data belong once they are collected). Although these threats are not unique to research using mobile technologies, there is a need to develop consent processes that actively engage individuals in their own privacy decision-making as much as possible [48,49].

### Conclusions

Overall, this study demonstrates that it is largely feasible to collect intensive longitudinal data on objective digital media use, simultaneously with accelerometry and EMA, from an economically and racially diverse sample of families with preschool-aged children. Although this study represents an initial improvement in objective measurement of digital media, additional measurement work is needed to advance the field to understand digital media use in the context of interpersonal dynamics and health.

## Appendix

Multimedia Appendix 1 Means and SDs of study measures (n=105).

## Abbreviations

CHAOS: Confusion, Hubbub, and Order Scale
EMA: ecological momentary assessment
RMSE: root mean square error
SDQ: Strengths and Difficulties Questionnaire
