# Supplementary material for: Feasibility of Measuring Screen Time, Activity, and Context Among Families With Preschoolers: Intensive Longitudinal Pilot Study
Source: JMIR Form Res. 2022 Sep 29;6(9):e40572. doi: 10.2196/40572 (PMC9562053; doi:10.2196/40572)
Supplement: Multimedia Appendix 1 [file formative_v6i9e40572_app1.docx]

|  | | | |
| --- | --- | --- | --- |
|  | **Mean** | **(SD)** | **Range** |
| **Child Measures** |  |  |  |
| Emotional Problems | 1.8 | 1.9 | 0 - 10 |
| Conduct Problems | 2.4 | 2.1 | 0 - 8 |
| Hyperactivity Problems | 4.6 | 2.8 | 0 - 10 |
| Peer Problems | 1.6 | 1.7 | 0 - 9 |
| Prosocial Problems | 7.9 | 1.7 | 3 - 10 |
| Total Problems | 10.3 | 6.1 | 1 - 30 |
| **Caregiver Measures** |  |  |  |
| Stress | 15.6 | 6.6 | 1 - 32 |
| Parenting Satisfaction | 16.2 | 2.6 | 9 - 21 |
| Anxiety | 10.9 | 3.5 | 6 - 20 |
| Depression | 6.6 | 4.4 | 0 - 21 |
| CHAOS ^a^ | 44.8 | 6.4 | 24 - 57 |
| Self-reported mobile phone use (n = 103) | 187.5 | 169.0 | 0 - 997 |
| ^a^ Lower CHAOS scores indicate higher household disorganization |  |  |  |
